# Fault-tolerant scheme for robotic manipulator—Nonlinear robust back-stepping control with friction compensation

**DOI:** 10.1371/journal.pone.0256491

**Published:** 2021-08-20

**Authors:** Khurram Ali, Adeel Mehmood, Jamshed Iqbal

**Affiliations:** 1 Department of Electrical and Computer Engineering, COMSATS University Islamabad, Pakistan; 2 Department of Computer Science and Technology, Faculty of Science and Engineering, University of Hull, Hull, United Kingdom; National Huaqiao University, CHINA

## Abstract

Emerging applications of autonomous robots requiring stability and reliability cannot afford component failure to achieve operational objectives. Hence, identification and countermeasure of a fault is of utmost importance in mechatronics community. This research proposes a Fault-tolerant control (FTC) for a robot manipulator, which is based on a hybrid control scheme that uses an observer as well as a hardware redundancy strategy to improve the performance and efficiency in the presence of actuator and sensor faults. Considering a five Degree of Freedom (DoF) robotic manipulator, a dynamic LuGre friction model is derived which forms the basis for design of control law. For actuator’s and sensor’s FTC, an adaptive back-stepping methodology is used for fault estimation and the nominal control law is used for the controller reconfiguration and observer is designed. Fault detection is accomplished by comparing the actual and observed states, pursued by fault tolerant method using redundant sensors. The results affirm the effectiveness of the proposed FTC strategy with model-based friction compensation. Improved tracking performance as well robustness in the presence of friction and fault demonstrate the efficiency of the proposed control approach.

## 1 Introduction

**Fig 1:**
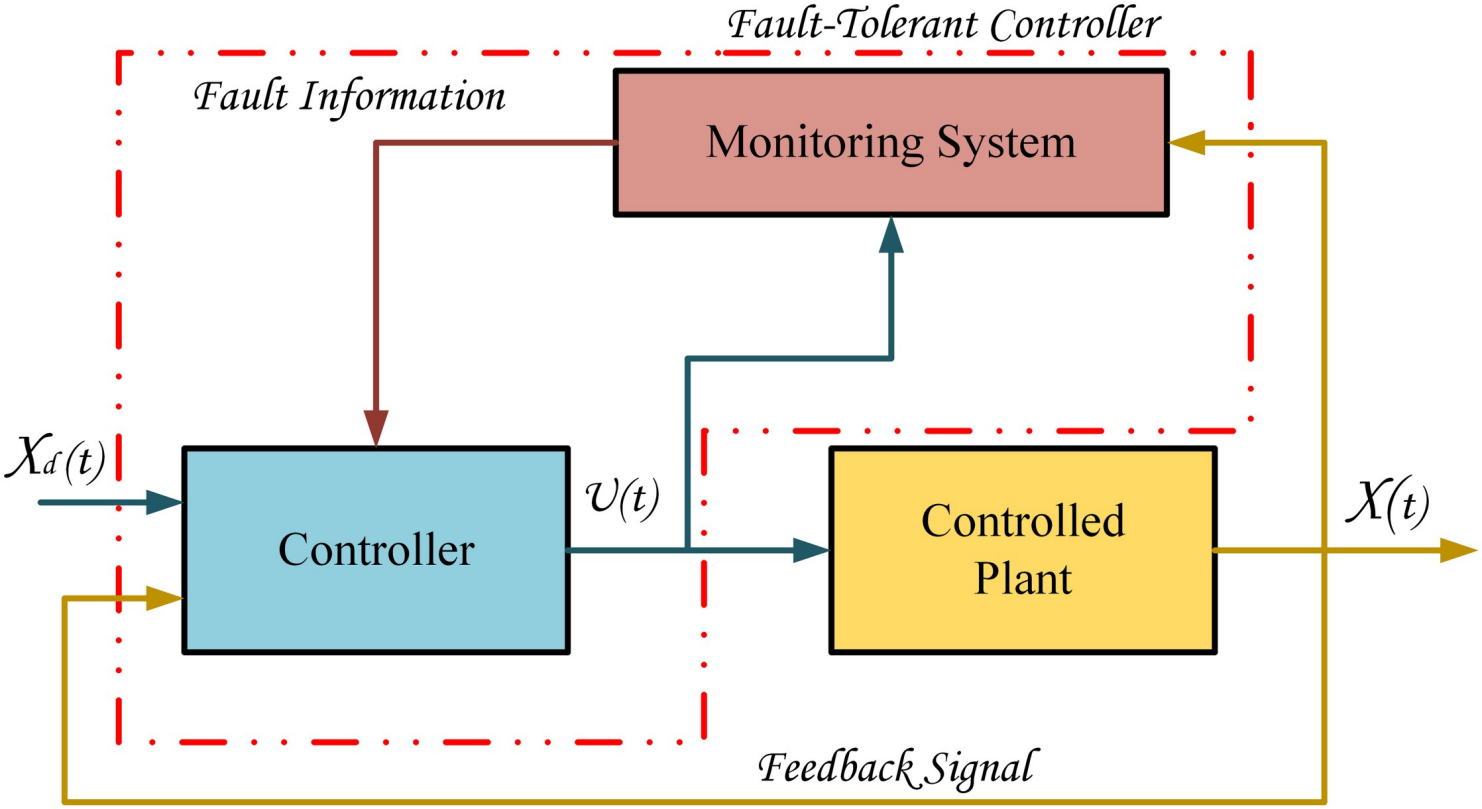
FTC architecture.

The state-of-the-art robots have been widely used in distinctive services for humankind. Robotic manipulators are currently installed in many industrial applications to perform various tasks [1]. The industries like medicine and surgery, pharmaceutical, military security, manufacturing and space exploration etc., are using industrial and service robots at different levels to facilitate human beings. Small industrial tasks including welding, assembling, and sorting can also be accomplished using robots [2]. With the enormous increase in robots applications in daily life, researchers are working on challenges which improve the performance of these robots. The increasing capability of performing complex tasks is making autonomous systems prompt to malfunction in accomplishing specific applications. To achieve the stability and better performance of system, control theory has been extensively established and applied to industrial processes [3]. The automated manipulators should be capable of completing their assigned task especially in the presence of one or sometimes more faults in their subsystems. Many FTC methods have been proposed with the ever-increasing requirements of upgrading the performance and reliability of a system [4]. The fundamental FTC architecture is described in the Fig 1.

**Fig 2:**
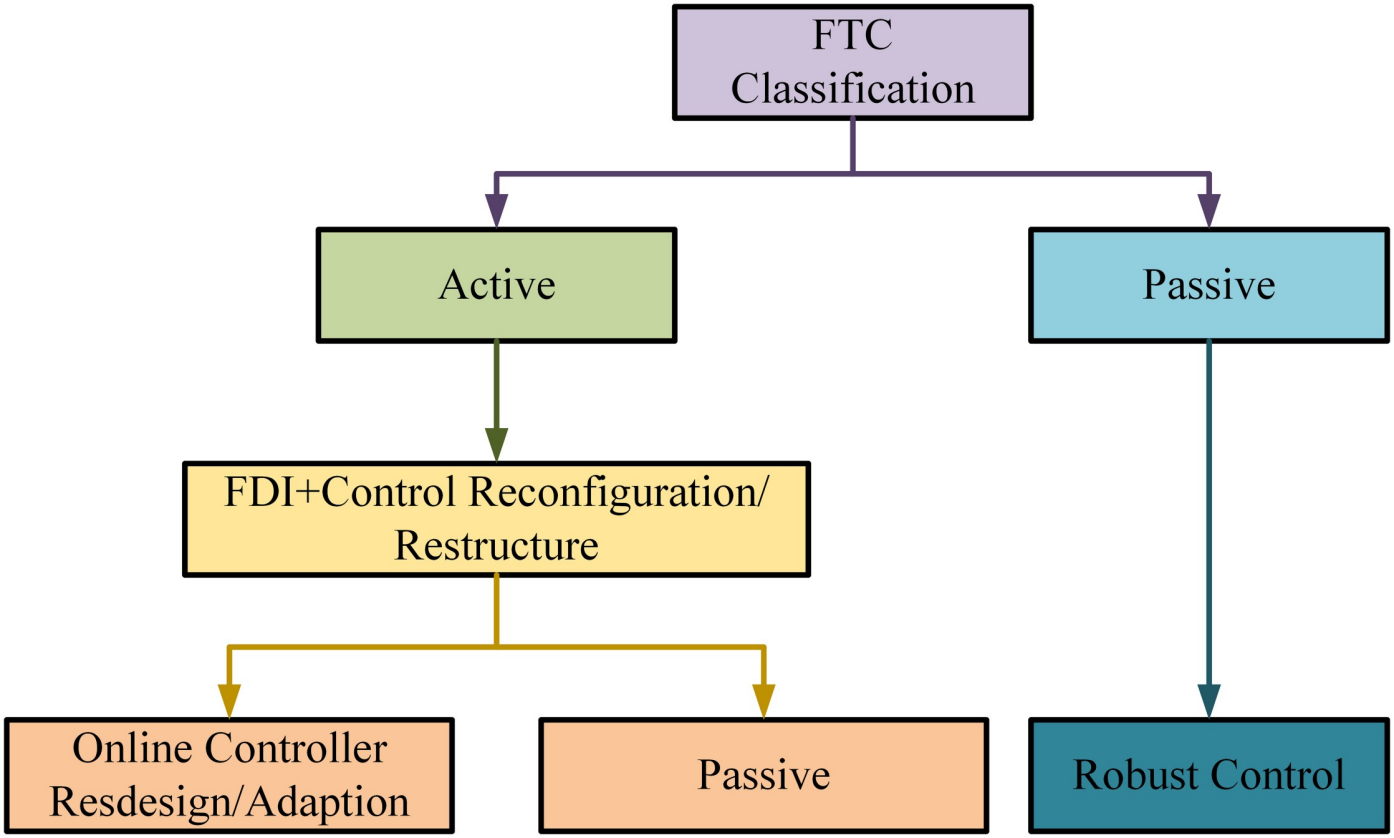
FTC classification.

FTC techniques have prime motive of detecting faults and preserving the performance in the existence of these faults. The typical fault occurrence can be in sensors and actuators of the robot manipulator [5]. Other reasons of faults can be in plant conditions, bad tuning of controller parameters, process abnormalities, damage in equipment and environmental changes. Stability, tracking, robustness and disturbance rejection are the prime objectives behind controller design [6]. FTC in robotic manipulator has the ability to detect faults and tolerate the failures [7]. Fault tolerance requires efforts at each stage and in all phases of system design. Numerous fault diagnosis (FD) methodologies for nonlinear robotic systems have been investigated previously. Mostly researchers have considered only the problems which are centered on mathematical models of plant. There are some non-mathematical challenges as well. FTC methods are majorly classified into two types [8, 9]. The FTC classification is briefly described in the Fig 2.

**Fig 3:**
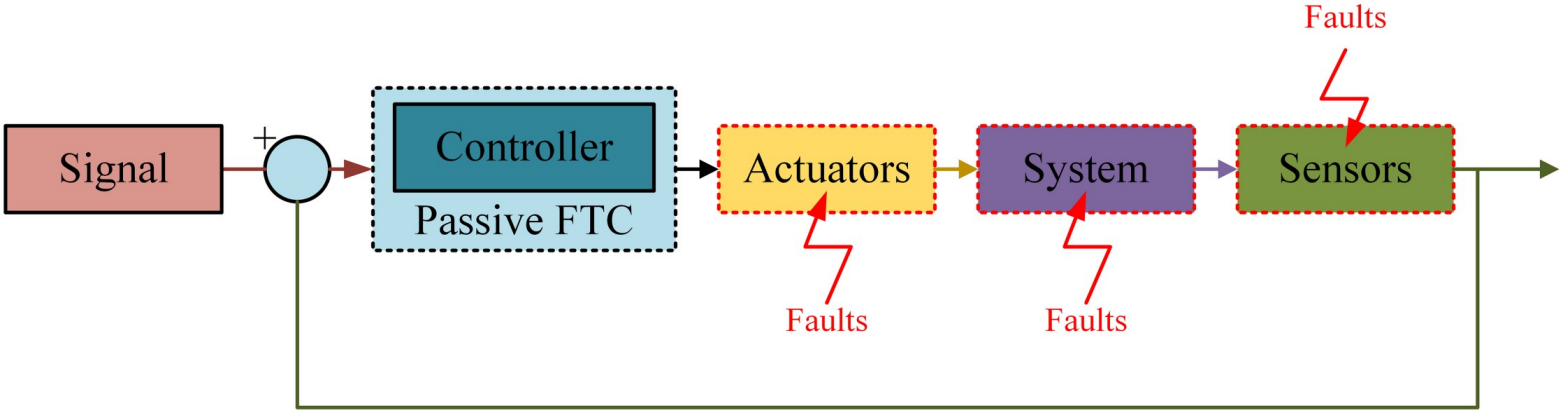
Passive FTC system.

The first type is known as Passive fault-tolerant control system (P-FTCS) and the other type is known as Active fault-tolerant control system (A-FTCS). In P-FTCS types of faults are not known to the control system [8]. In Passive methods close loop controller are designed to ensure stability and performance in the presence of operational components with fault [10]. Passive methods include adaptive control and robust control. In robust passive method controller is designed such that system is insensitive to the faults mainly of sensors and actuators [11]. The Riccati equations to design sensor fault based LQR controller for linear systems is presented in [12]. In P-FTCS techniques, one controller is employed for the standard case and the fault case where it is not necessary to identify the existence of fault [13, 14]. These articles consider the passive means of fault tolerance established on various robust control design techniques. Fig 3 describes the P-FTCS system in the form of block diagram.

**Fig 4:**
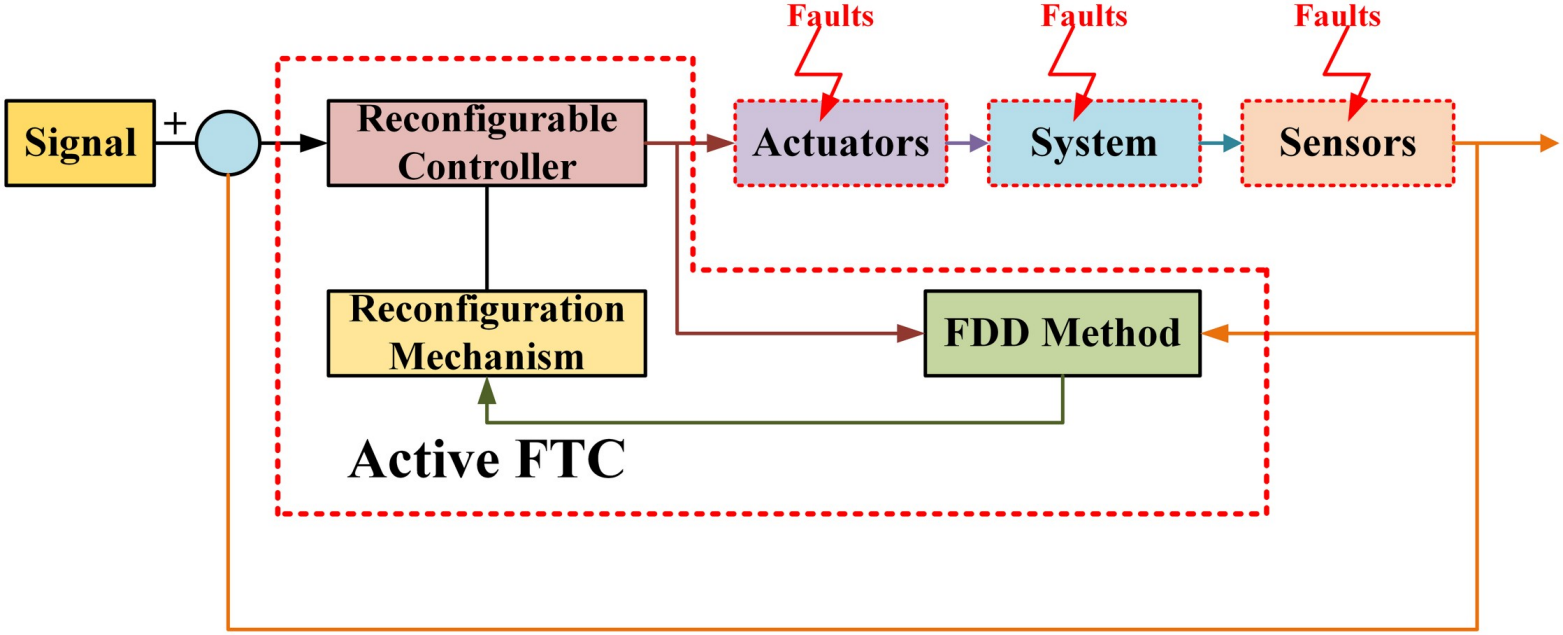
Active FTC system.

Moreover, fault-tolerance is achieved in P-FTCS methods by defining faults as disturbances in the system, allowing for the configuration of a robust controller. Various types of systems are taken into account with the primary goal of fault compensation without the use of a prior detection algorithm. The key idea is to assume of a fault as a bounded uncertainty that can be compensated while using a nominal control system. The block diagram presentation of of A-FTCS method is presented in Fig 4. The controller is designed on the basics of fault information in the A-FTCS, and the first step is fault detection to acquire fault information [15]. An A-FTCS technique is suggested for additive sensor faults in [16]. Firstly, observer is used for fault detection and when the fault is detected then fault isolation observers are activated to control the faulty sensor. If the fault is a recognizable then the control objective stays the same; however, if the fault is non-recognizable, the goal changes, the controller ensures the converges of healthy output to the desired point.

**Fig 5:**
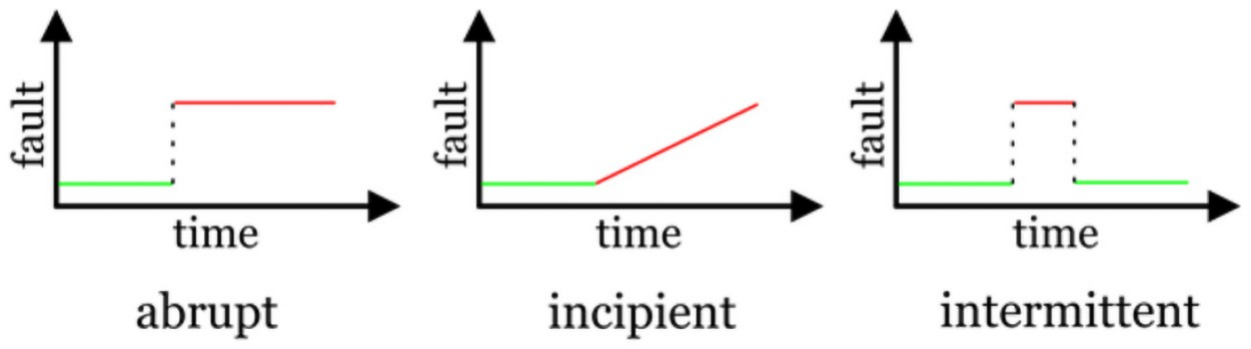
Types of fault.

Based on fault tolerant observer an A-FTCS is also proposed for rail friction drive with sensor disconnection faults [17]. In [18] FTC for ship propulsion benchmark with estimated measured feedback variables is used. Another article proposed sensor fault-based FTC for multiple input multiple output non-linear dynamic systems [19]. This is a robust method with bounded uncertainties. In [20] an effective Fault tolerant system (FTS) is designed which is basically named as Adaptive fault tolerant control System (AD-FTCS). Estimator works on self-adjustable design idea and the theory of active method is straightforward that when a fault occurs in a system, the system deviates from its nominal operating point to a faulty one [21]. The proposed system in [22] uses adaptive estimation and control strategies for nonlinear time invariant systems. Neural and fuzzy systems have an ability to accurately approximate to any continuous function. To deal with nonlinearities; the idea of function approximation has been used in adaptive control [23]. In [24] adaptive control is implemented on jet engine to compensate the sensor fault. Adaptive method for fault detection and identification in linear time invariant (LTI) systems is proposed in [25]. In the FTC system, faults are identified according to their location of occurrence in a system. Classification of faults are done on the basics of time characteristics as presented in Fig 5.

Instantaneous changes in output with respect to time are known as abrupt faults, which more often occurs due to faulty or hardware damage. Typically, Abrupt faults in a system are very severe. They affect the stability of the system and its performance, and moreover such results need quick and speedy reaction by the FTC system. Incipient faults are initial defects which characterizes slow changes in parameters over time, often due to aging. Incipient faults are more tough to detect and distinguish due to their slow time-based characteristics, nevertheless they are similarly less severe. Intermittent faults are defects which occur and disappear frequently, for example, due to partially damaged wiring. A linear system with actuator and sensor actuator faults can be represented as Eq (1)

$$\begin{array}{c} \begin{array}{cc} \dot{q} & =A_{i}q+B_{i}\left(u+F_{t}\left(u,q,t\right)\right)\\ y & =C_{i}q+F_{s}\left(u,q,t\right) \end{array}\} \end{array}$$ (1)

Where *q* ∈ ℜ^*n*x1^ represent state vectors, *y* ∈ ℜ^*m*x1^ represent output vectors and *u* ∈ ℜ^*p*x1^ represent input vectors. *F*_*t*_ ∈ *R*^*p*x1^ show the actuator fault added to the input and the sensor fault *F*_*s*_ ∈ *R*^*m*x1^ is added to the output. The following are the major attributions of this paper, summarized as follows:

- Firstly, for the purpose of implementing the robust control algorithm, a five DoF serial link Autonomous Articulated Robotic Educational Platform (AUTAREP) manipulator has been modeled by considering the dynamic LuGre friction model.
- In the initial stage, nominal control law is formulated to enhance robustness using back-stepping technique that can converges the given Lyapunov candidate function to a finite-value.
- For actuator FTC, an adaptive back-stepping technique is employed for fault estimation and tolerance. In the case of sensor FTC, an observer and nominal controller are designed whereas as residuals are generated for fault indication and switching of sensors.
- Moreover, the Lyapunov technique is utilized to rigorously analyze the stability and durability of robotic manipulator. The proposed FTC based approach is finally validated in simulation in MATLAB/Simulink environment with incipient, intermittent and abrupt faults to characterize the control performance.

Rest of the article is organized as follows; Section 1 demonstrates the mathematical modelling by considering the dynamics of robot manipulator using dynamic LuGre friction model. In Section 2, the nominal back-stepping control law is designed along with sensor and actuator FTC. After designing the control law, in section 3, outcomes of the control design and FTC scheme have been analyzed. At the end, the article is concluded in section 4.

## 2 Modeling

**Fig 6:**
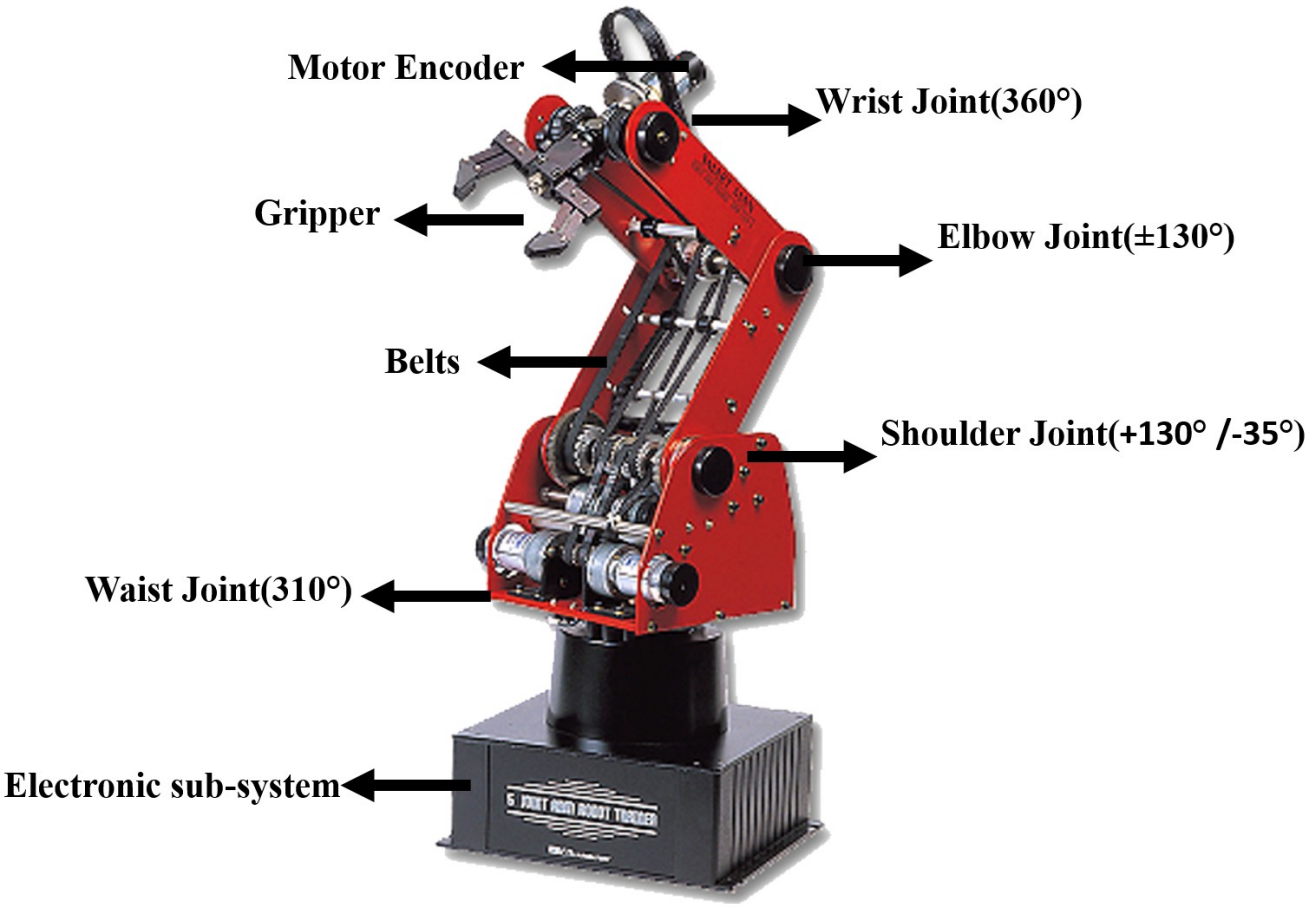
AUTAREP manipulator ED-7220C showing various joints with their Range Of Motion(ROM).

In this research, ED-7220C robot arm is used which is an AUTAREP [26] as shown in Fig 6.

The robotic arm has five revolute joints (wrist, elbow, shoulder, waist or base joints) with five DoF. Each joint of the manipulator is actuated with a DC servo motor having an optical encoder for position feedback. A single motor is used to move each joint except wrist joint where for pitch and roll involves two motors. The generalized manipulator’s dynamic equation for a n-DoF system is given by

$$\begin{array}{c} \tau_{f}=M\left(q_{i},{\dot{q}}_{i}\right)\;{\ddot{q}}_{i}\;+\;C_{c}\left(q_{i},{\dot{q}}_{i}\right)\;+\;G\left(q_{i}\right)\;+\;F_{r}\left({\dot{q}}_{i}\right) \end{array}$$ (2)

where *M*(*q*_*i*_) ∈ ℜ^*n*×*n*^ is the mass/inertial matrix, *C*_*c*_(*q*_*i*_) ∈ ℜ^*n*^ represents the centripetal and coriolis forces, *G*(*q*_*i*_) ∈ ℜ^*n*^ is the gravitational matrix, the term *τ*_*f*_ ∈ ℜ^*n*^ is the vector input torque applied to the joints of the robot. $F_{r}\left(\dot{q}\right)\in {ℜ}^{n}$ represents frictional forces, friction is one of main causes of undesirable system response because it causes hysteresis and limit cycles and hence degrades its performance [27, 28]. In the literature, a number of dynamic friction models have been suggested, including the Dahl model [29] and LuGre model [30] etc. The LuGre friction model is based on the dynamic Dahl friction model, which is an integrated dynamic model of friction. The Stribeck effect and viscous friction are included in the LuGre model, which is given as

$$\begin{array}{c} F_{r}=\sigma_{0}\;z+\sigma_{1}\;\dot{z}+\;f\left(\omega \right) \end{array}$$ (3)

where *ω* is the velocity between the two surfaces in contact, *z* is the internal friction state, *F*_*r*_ is the predicted friction force, *σ*_0_ is stiffness coefficient, *σ*_1_ is damping coefficient and *σ*_2_ is viscous friction coefficient, typically, *f*(*ω*) = *σ*_2_ *ω* in Eq (3). The dynamics of friction state $\dot{z}$ can be defined as

$$\begin{array}{c} \dot{z}=\omega -\sigma_{0}\;\frac{\left|\;\omega \;\right|}{g\;(\omega )}\;z \end{array}$$ (4)

where *g* (*ω*) in Eq (4) is given by

$$\begin{array}{c} g\;\left(\omega \right)=(\;F_{c}+(\;F_{s}-F_{c}\;)\;\exp^{-\;(\;|\omega /\omega_{s}|)}\;) \end{array}$$ (5)

where *F*_*s*_ corresponds to the static friction, *F*_*c*_ is the coulomb friction and *ω*_*s*_ is the sliding speed coefficient. The *ω*_*s*_ is also called as stribeck velocity. The aim of FTC is to compensate for the deficiency caused by a fault, as well as to maintain system stability and recover fault-free results. The dynamics of a fault-free re-configurable manipulator with *n* DoF is described by using Lagrangian formulation, i.e.,

$$\begin{array}{c} \tau_{f}\;=M\left(q_{i},{\dot{q}}_{i}\right)\;{\ddot{q}}_{i}\;+\;C_{c}\;\left(q_{i},\;{\dot{q}}_{i}\right)\;+G\;\left(q_{i}\right)+\;F_{r}\;\left({\dot{q}}_{i}\right)\;+⋎\;\left(t-T_{f}\right)\;\Phi \;\left(t\right) \end{array}$$ (6)

where ⋎(*t* − *T_f_*) presents the time profile of the faults and *T*_*f*_ is the time of occurrence of the faults. Φ(*t*) ∈ ℜ^*n*×1^ is a vector composed of actuator faults and component faults. ⋎(*t* − *T_f_*) is a step function defined as

$$\begin{array}{c} ⋎\;\left(t-T_{f}\right)=\{\begin{array}{cc} 0 & t<T_{f}\\ 1 & t\geq T_{f} \end{array} \end{array}$$ (7)

The objective of this research is to design a reconfigurable FTC strategy for the mechanical system Eq (2) that guarantees the same control results as obtained from the nominal control law in face of actuator faults and uncertain dynamics. In the position control, the Eq (6) with faults for n-DoF robot manipulator can be rewritten as

$$\begin{array}{c} {\ddot{q}}_{i}=\;M^{-1}\;\left(\tau_{f}-C_{c}\;\left(q_{i},{\dot{q}}_{i}\right)\;-G\;\left(q_{i}\right)-F_{r}\;\left({\dot{q}}_{i}\right)-⋎\left(t-T_{f}\right)\;\Phi \left(t\right)\right) \end{array}$$ (8)

Consider *F_t_* = ⋎(*t* − *T_f_*)Φ(*t*), the dynamics of AUTAREP manipulator is discussed below. Let *q*_*i*1_ is the position vector, *q*_*i*2_ is the velocity vector and *q*_*i*3_ is internal friction state. Thus the system equations can be written as:

$$\begin{array}{c} {\ddot{q}}_{i}=\;M^{-1}(\;\tau_{fi}-\left(\;C_{c}\;q_{i2}+G\;q_{i1}+\sigma_{i0}\;q_{i3}++\sigma_{i2}\;q_{i2}\;+\sigma_{i1}\;{\dot{q}}_{i3}\;\right)-F_{ti} \end{array}$$ (9)

where *i* = 1, 2, 3, 4, 5, 6 in Eq (9).

**Remark**: The objective of this research is to develop a control input (*τ*_*fi*_) in such a manner that the system can offer excellent tracking performance especially in the presence of faults, uncertainties and disturbances.

## 3 Control design

The control design methodology has been implemented in three phases; In the initial phase, nominal control law is designed using back-stepping technique. In the second phase, the adaptive back-stepping control approach is used for estimation of fault. The third phase establishes the rules and regulations for the sensor faults compensation when faults of sensor are identified from the residuals.

### 3.1 Nominal control

The back-stepping controller is designed to achieve the nominal performance for a non-linear robot manipulator. In the scenario of fault, the nominal control is modified to preserve performance. Passive FTC is accomplished by designing the back-stepping control method [31] and considering the fault as a bounded uncertainty [32]. The design controller can be used as nominal control for A-FTCS design, further for the on-line faults estimation and tolerance is performed by modifying this nominal controller. The control law is derived by reorganizing the state equations of robot manipulator dynamic model and it is converted into subsystems. Henceforth, every joint of robot manipulator has a specific state equations set. The subsystems model of robot manipulator are characterized as:

$$\begin{array}{c} \begin{array}{cc} {\dot{q}}_{1} & =q_{2}\\ {\dot{q}}_{2} & =f_{1}\left(q_{1},q_{2},q_{3},....q_{6}\right)-m_{11}^{-1}F_{r1}+m_{11}^{-1}\tau_{f1}\\ {\dot{q}}_{3} & =q_{4}\\ {\dot{q}}_{4} & =f_{2}\left(q_{1},q_{2},q_{3},....q_{6}\right)-m_{22}^{-1}F_{r2}+m_{22}^{-1}\tau_{f2}\\ {\dot{q}}_{5} & =q_{6}\\ {\dot{q}}_{6} & =f_{3}\left(q_{1},q_{2},q_{3},....q_{6}\right)-m_{33}^{-1}F_{r3}+m_{33}^{-1}\tau_{f3} \end{array}\} \end{array}$$ (10)

The error dynamics of joint positions *q*_1_ is defined by error vector *e* where it is a vector of *n* rows.

$$\begin{array}{c} e=q_{1}-q_{d} \end{array}$$ (11)

By taking derivative of Eq (11)

$$\begin{array}{c} \dot{e}=q_{2}-{\dot{q}}_{d} \end{array}$$ (12)

Similarly for n-joints, the position state for *i*^*th*^ joint is *q*_2*i*−1_ for 1 ≤ *i* ≤ 3 and d the desired trajectory for respective joints is *q*_*di*_. The dynamics of tracking error *e*_*i*_ is given by:

$$\begin{array}{c} e_{i}=q_{2i-1}-q_{di} \end{array}$$ (13)

The stabilizing function $\alpha (q_{1},q_{d},{\dot{q}}_{d})$ of the system for virtual control with the error dynamics is given by

$$\begin{array}{c} z=q_{2}-\alpha \left(q_{1},q_{d},{\dot{q}}_{d}\right) \end{array}$$ (14)

where *z* is the virtual state in Eq (14). The *α*_*i*_ stabilizing function for the *i*^*th*^ joint is given by,

$$\begin{array}{c} \alpha_{i}\left(q_{2i-1},q_{di},{\dot{q}}_{di}\right)={\dot{q}}_{di}-k_{i}e_{i} \end{array}$$ (15)

where *k*_*i*_ is a positive design parameter. By replacing the Eq (13) into Eq (15) and then its time derivative can be characterized as

$$\begin{array}{c} {\dot{\alpha }}_{i}\left(q_{2i-1},q_{di},{\dot{q}}_{di}\right)=-k_{i}q_{2i}+k_{i}{\dot{q}}_{di}+{\ddot{q}}_{di} \end{array}$$ (16)

By considering the *z*_*i*_ is the virtual control deviation of *q*_2*i*_ to its desired value of *α*_*i*_

$$\begin{array}{c} \begin{array}{cc} z_{i} & =q_{2i}-\alpha_{i}\left(q_{2i-1},q_{di},{\dot{q}}_{di}\right)\\ {\dot{z}}_{i} & =m_{ii}^{-1}\tau_{fi}-m_{ii}^{-1}F_{ri}+f_{i}+k_{i}q_{2i}-k_{i}{\dot{q}}_{di}-{\ddot{q}}_{di} \end{array}\} \end{array}$$ (17)

The foremost step in controller design is to stabilize the system by using lyapunov function. The stability of system is ensured by defining Lyapunov function in a such way that *V*_*ρ*_(*q*) > 0 ∀*q* ≠ 0 and the lyapunov function is given by Eq (18).

$$\begin{array}{c} V_{\rho }\left(e,z\right)=\frac{1}{2}z^{T}z+\frac{1}{2}e^{T}e \end{array}$$ (18)

However, the torque is anticipated input which make sure Lyapunov stability in the system and the derivative of Eq (18) is given by

$$\begin{array}{c} {\dot{V}}_{\rho }\left(e_{i},z_{i}\right)=\sum_{i=1}^{3}z_{i}{\dot{z}}_{i}+\sum_{i=1}^{3}e_{i}{\dot{e}}_{i} \end{array}$$ (19)

where $\dot{e}$ in Eq (19) is given by

$$\begin{array}{c} \dot{e}=z_{i}-k_{i}e_{i} \end{array}$$ (20)

Substituting Eqs (17) & (20) in the Eq ((19)) and rewriting Eq (19)

$$\begin{array}{c} \begin{array}{cc} {\dot{V}}_{\rho }\left(e_{i},z_{i}\right) & =\sum_{i=1}^{3}z_{i}\;(m_{ii}^{-1}\left(\tau_{fi}-F_{ri}\right)+k_{i}q_{2i}-k_{i}{\dot{q}}_{di}-{\ddot{q}}_{di}\\ & \;\;\;+f_{i})+\sum_{i=1}^{3}e_{i}\;\left(z_{i}-k_{i}e_{i}\right) \end{array} \end{array}$$ (21)

$$\begin{array}{c} c_{i}z_{i}=-\left(e_{i}+m_{ii}^{-1}\left(\tau_{fi}-F_{ri}\right)+k_{i}\;q_{2i}-k_{i}\;{\dot{q}}_{di}-{\ddot{q}}_{di}+f_{i}\right) \end{array}$$ (22)

The input command *τ*_*i*_ for *i*_*th*_ joint is given by Eq (23)

$$\begin{array}{c} \tau_{fi}=m_{ii}\left(-c_{i}\;z_{i}-e_{i}-k_{i}\;q_{2i}+k_{i}\;{\dot{q}}_{di}+{\ddot{q}}_{di}+F_{ri}-f_{i}\right) \end{array}$$ (23)

The closed loop system of robot manipulator model is globally asymptotically stable closed loop system for the given input torques. Therefore, the Lyapunov energy fuction derivative is negative definite and the error function in the finite time converges to zero for the $\dot{V_{\rho }}\left(q\right)<0\;\forall \;q\neq 0$,

$$\begin{array}{c} {\dot{V}}_{\rho }\;\left(e_{i},z_{i}\right)=-\;e_{i}^{T}\;k_{i}\;e_{i}-z_{i}^{T}\;c\;z_{i}\leq \;0 \end{array}$$ (24)

where *k*_*i*_ presents the relationship to the controller gain for 1 ≤ *i* ≤ 3. It is the controller gain (*k*_*i*_) parameter which is required to be greater than zero to ensure stability and finite time convergence.

**Remark**: By rearranging the state equations of the manipulator dynamic model and transforming into subsystems, the control input torque (*τ*_*fi*_) is determined to enhance the tracking performance. Moreover, positive definite Lyapunov candidate function is used for stability analysis of the system and its derivate is ensured to be negative definite which guarantees stability. In case of any positive design parameters (*k*_*i*_ > 0, *c* > 0), the system is assured to be uniformly bounded and globally stable.

### 3.2 Actuator fault tolerance

**Fig 7:**
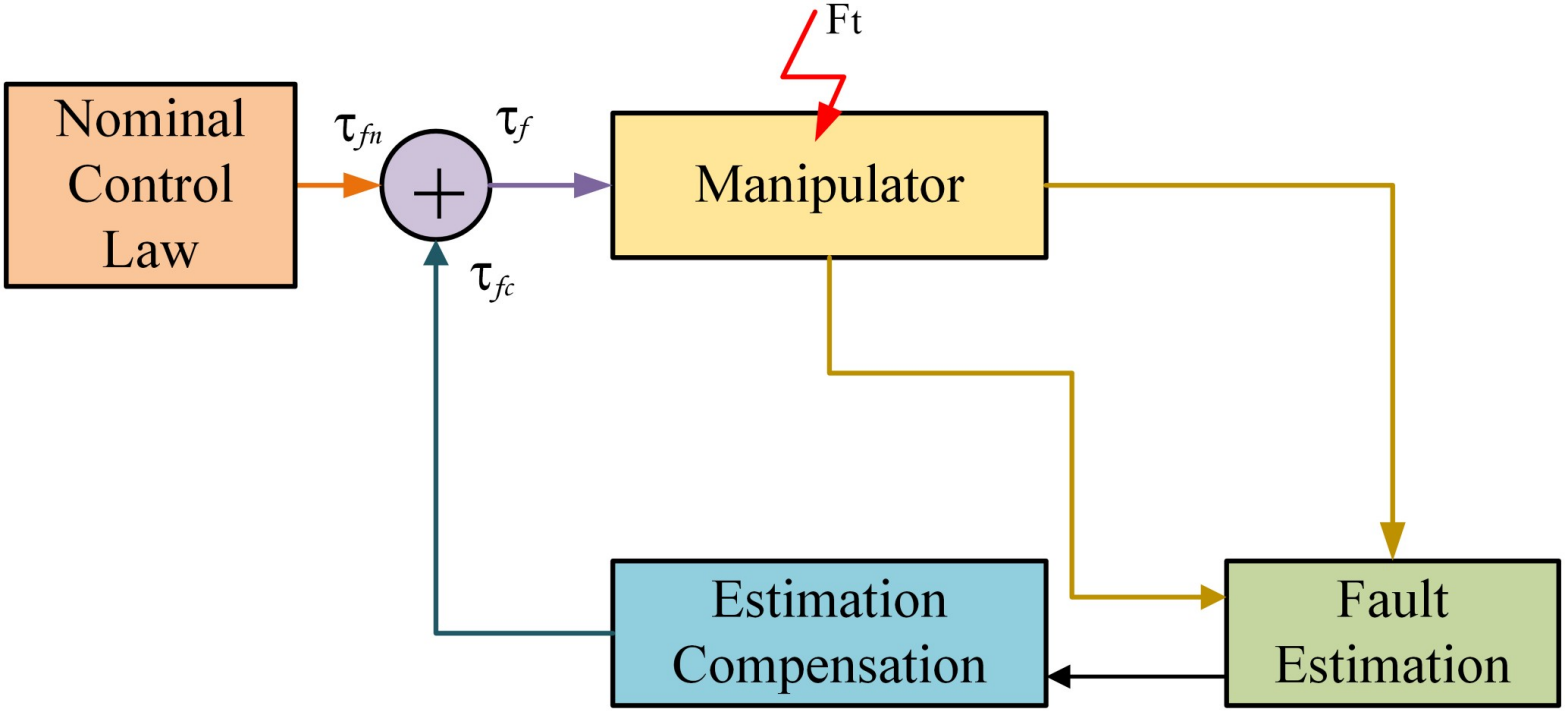
Actuator FTC design technique.

An actuator fault is a kind of failure affecting behavior of the system inputs. There are lot of reasons for occurrence of actuator fault like material aging or due to abnormal procedure and operation. The failures in the system due to actuators might drastically change and alter system behavior and resulting in system instability. In the suggested active FTC design methodology, the adaptive back-stepping strategy is adopted for the estimation of fault. There is an extra term adds up to input for estimation of fault as well as for compensate the fault in a system. The algorithm of active FTC technique is demonstrated in the Fig 7.

The fault term of actuator is included in the motors torque of robot manipulator. Let the vector of fault added in input is *F*_*t*_, then its system modeling is characterized by:

$$\begin{array}{c} \ddot{q}=M^{-1}\left(\tau_{f}-C_{c}\left(q_{i},\dot{q_{i}}\right)\dot{q_{i}}-G\left(q_{i}\right)-F_{r}\left(\dot{q_{i}}\right)\right)+M^{-1}F_{t} \end{array}$$ (25)

For the same plant model of 3 subsystems, fault is added in the torques and active FTC approach to fault estimation and controller reconfiguration is used to compensate the fault in any of the actuator. Let the fault profile addition *i*^*th*^ joint is *F*_*ti*_ and its estimate is ${\hat{F}}_{ti}$. The actual fault and estimated fault profile ${\tilde{F}}_{ti}$ difference ought to converge in order to ensure the stability. The Lyapunov function candidate is defined in Eq (26).

$$\begin{array}{c} V_{\rho }\left(q,\tilde{F_{t}}\right)=\frac{1}{2}e^{T}e+\frac{1}{2}z^{T}z+\sum_{i=1}^{n}\left(1/\gamma_{i}\right){\tilde{F}}_{ti}^{2} \end{array}$$ (26)

To obtain the control input torque terms established on fault profile estimation, the further simplification of Lyapunov is carried out. The derivative of Eq (26) is given by

$$\begin{array}{c} {\dot{V}}_{\rho }\left(q,\tilde{F_{t}}\right)=\sum_{i=1}^{n}e_{i}{\dot{e}}_{i}+\sum_{i=1}^{n}z_{i}{\dot{z}}_{i}+\sum_{i=1}^{n}\left(1/\gamma_{i}\right){\tilde{F}}_{ti}{\dot{\tilde{F}}}_{ti} \end{array}$$ (27)

Let the fault of actuator have been added to the joints of robot manipulator like waist joint as well to shoulder joint or both, therefore Eq (27) will be

$$\begin{array}{c} {\dot{V}}_{\rho }\left(q,\tilde{F_{t}}\right)=e^{T}\dot{e}+z^{T}\dot{z}+\left(1/\gamma_{1}\right){\tilde{F}}_{t1}\left({\dot{\tilde{F}}}_{t1}\right)+\left(1/\gamma_{2}\right){\tilde{F}}_{t2}{\dot{\tilde{F}}}_{t2} \end{array}$$ (28)

where for *i* = 1, 2.

$$\begin{array}{c} {\dot{z}}_{1}=F_{t1}\;M^{-1}\left(1,1\right)+\;M^{-1}\left(1,1\right)\;\left(\tau_{f1}-C_{c1}-\;G_{1}+F_{r1}\right)+k_{1}\;\left(q_{2}-{\dot{q}}_{d1}\right)-\;{\ddot{q}}_{d1}\;+\;e_{1} \end{array}$$ (29)

$$\begin{array}{c} {\dot{z}}_{2}=F_{t2}\;M^{-1}\left(2,2\right)+M^{-1}\left(2,2\right)\;\left(\tau_{f2}-C_{c2}-\;G_{2}+F_{r2}\right)+k_{2}\;\left(q_{4}-{\dot{q}}_{d2}\right)-{\ddot{q}}_{d2}\;+\;e_{2} \end{array}$$ (30)

where *M*^−1^(1, 1) and *M*^−1^(2, 2) are given in Eqs (31) and (32) respectively.

$$\begin{array}{c} M^{-1}\left(1,1\right)=-\frac{m_{22}}{m_{22}m_{11}} \end{array}$$ (31)

$$\begin{array}{c} M^{-1}\left(2,2\right)=-\frac{m_{11}}{m_{22}m_{11}} \end{array}$$ (32)

The motor torques are well-defined in such way that Lyapunov function derivate is negative definite in Eq (28). The total torque input is the addition of *τ*_*fc*_ the compensation torque and nominal torque input *τ*_*fn*_ term. The Compensational term of total torque is articulated with fault profile which is estimated for the associated joint. Henceforth for *i* = 1, 2.

$$\begin{array}{c} \tau_{fi}=\tau_{fni}+\tau_{fci} \end{array}$$ (33)

The equations of torque for robot manipulator waist joint are given below,

$$\begin{array}{c} \tau_{fc1}=-\Gamma_{1}{\hat{F}}_{t1} \end{array}$$ (34)

$$\begin{array}{c} \tau_{fn1}=m_{11}\left(k_{1}\left({\dot{x}}_{d1}-x_{2}\right)+{\ddot{x}}_{d1}-e_{1}-c_{1}z_{1}\right)+C_{c1}+G_{1}+F_{f1} \end{array}$$ (35)

The robot manipulator shoulder joint equations are

$$\begin{array}{c} \tau_{fc2}=-\Gamma_{2}{\hat{F}}_{t2} \end{array}$$ (36)

$$\begin{array}{c} \tau_{fn2}=m_{22}\left(k_{2}\left({\dot{x}}_{d2}-x_{4}\right)+{\ddot{x}}_{d2}-e_{2}-c_{2}z_{2}\right)+C_{c2}+G_{2}+F_{f2} \end{array}$$ (37)

where Γ_1_ as well as Γ_2_ are design parameters having positive value. The Eq (28) is further simplified for fault estimation. In the specific time interval the assumed fault should have constant derivative. Thus, the faults of actuator are supposed to have meet the following requirements.

$$\begin{array}{c} {\dot{\tilde{F}}}_{t1}=-{\dot{\hat{F}}}_{t1} \end{array}$$ (38)

$$\begin{array}{c} {\dot{\tilde{F}}}_{t2}=-{\dot{\hat{F}}}_{t2} \end{array}$$ (39)

$$\begin{array}{c} z_{1}M^{-1}\left(1,1\right){\tilde{F}}_{t1}-\left(1/\gamma_{1}\right){\tilde{F}}_{t1}{\dot{\hat{F}}}_{t1}=0 \end{array}$$ (40)

$$\begin{array}{c} z_{2}M^{-1}\left(2,2\right){\tilde{F}}_{t2}-\left(1/\gamma_{2}\right){\tilde{F}}_{t2}{\dot{\hat{F}}}_{t2}=0 \end{array}$$ (41)

From above equations the actuator faults estimation of waist joint and shoulder joint are given below

$$\begin{array}{c} {\dot{\hat{F}}}_{t1}=\gamma_{1}z_{1}M^{-1}\left(1,1\right) \end{array}$$ (42)

$$\begin{array}{c} {\dot{\hat{F}}}_{t2}=\gamma_{2}z_{2}M^{-1}\left(2,2\right) \end{array}$$ (43)

**Remark**: The suggested approach for the actuator FTC is provided in this instance when the robot manipulator states are observable. The control input signal is the sum of the compensation torque (*τ*_*f*_ *c*) and the nominal torque (*τ*_*f*_ *n*). The compensation torque is coupled with the expected fault profile for the corresponding joint. The estimation of actuators fault (waist and shoulder) are described in Eqs (42) and (43).

### 3.3 Sensor fault tolerance

**Fig 8:**
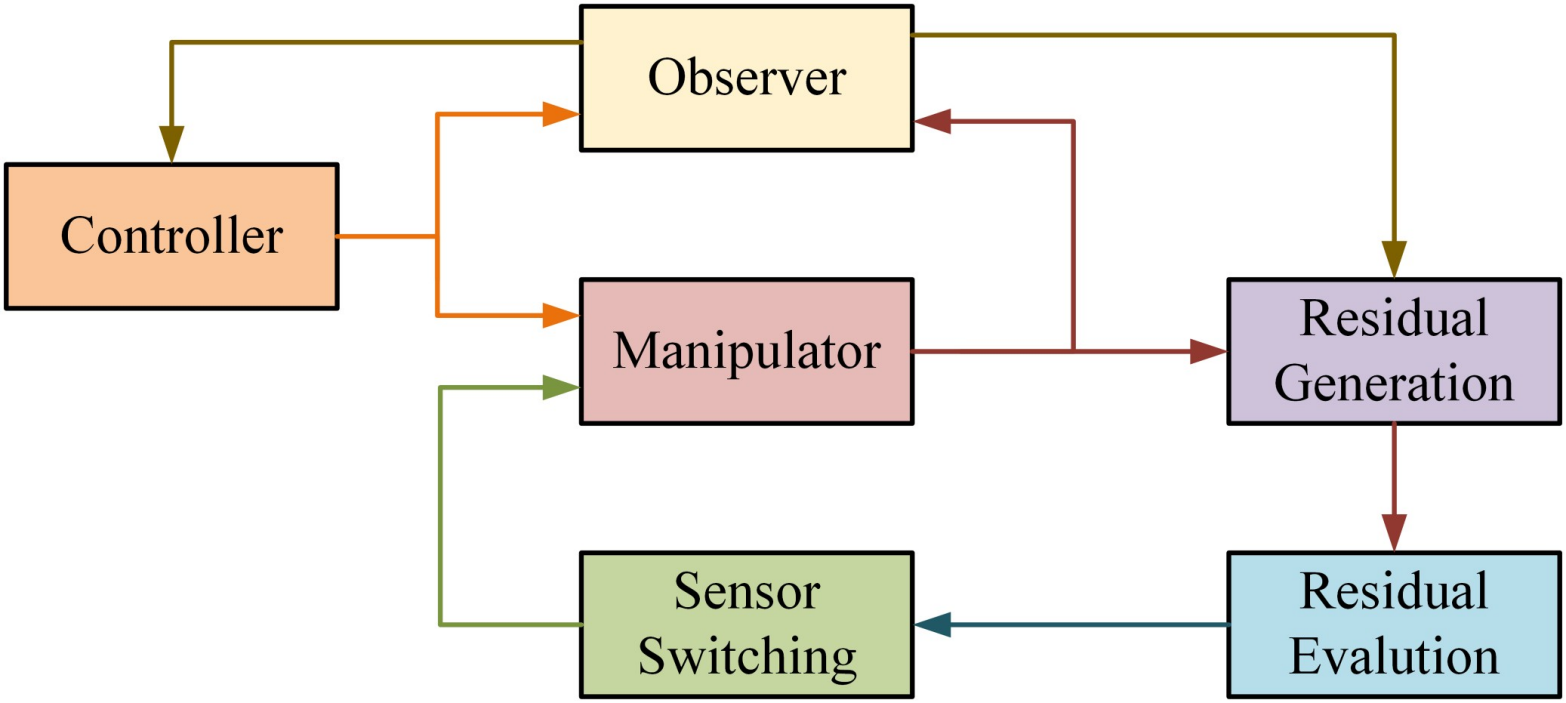
Sensor FTC.

The sensor faults occurs due to the incorrect reading of the system from the equipped sensors. The overall fault of sensor generates data and information which is not associated to measured physical parameter value. The Fault in the system is due to multiple causes like damaged wires or missed contact with the surfaces etc. In the suggested methodology the active approach is considered for sensor FTC. This methodology is justified by model free design methods and model reference combination. Initially, the estimation of states is achieved by utilizing the observer design method. The objective of an observer in control theory is to get the state estimation from input measurements and output of the robot manipulator in interval of predictable time. The comparison of actual positions and the positions estimated by the observers are used to generate residuals. These residual are further passed on to the decision making block for evaluation. This gives an idea about the presence of fault. Thus fault estimation block estimates the type and magnitude of the fault and the Robust/nominal control law (Back-stepping) is reconfigured to adjust the response in the presence of fault. For simulation purpose firstly a super twisting observer is designed. The observer takes the position from the actual model and estimates the velocity. The difference between the estimated position and the position at the Sensor output of respective joint constitutes the error. Fig 8 demonstrates the proposed approach. In this paper, the actuator FTC proposed in subsection 3.2 does not involve state observer as illustrated in Fig 7. On the other hand, the methodology adopted for sensor FTC in subsection 3.3 is an observer-based approach involving the design of a super twisting observer Fig 8.

For simulation objective firstly a super twisting observer is designed. The designed observer for sensor FTC takes the position from the actual model and estimates the velocity. The difference between the estimated position and the position at the sensor output of respective joint constitutes the error. The super twisting algorithm based observer is designed for dynamic model of robot manipulator and observer for the subsystem has the structure provide by

$$\begin{array}{c} \begin{array}{cc} {\dot{\hat{q}}}_{1} & ={\hat{q}}_{2}+\rho_{1}\\ {\dot{\hat{q}}}_{2} & ={\hat{f}}_{1}\left(q_{1},{\hat{q}}_{2},q_{3},....{\hat{q}}_{6}\right)+m_{11}^{-1}\left(\tau_{f1}-F_{r1}\right)+\rho_{2}\\ {\dot{\hat{q}}}_{3} & ={\hat{q}}_{4}+\rho_{3}\\ {\dot{\hat{q}}}_{4} & ={\hat{f}}_{2}\left(q_{1},{\hat{q}}_{2},q_{3},....{\hat{q}}_{6}\right)+m_{22}^{-1}\left(\tau_{f2}-F_{r2}\right)+\rho_{4}\\ {\dot{\hat{q}}}_{5} & ={\hat{q}}_{6}+\rho_{5}\\ {\dot{\hat{q}}}_{6} & ={\hat{f}}_{3}\left(q_{1},{\hat{q}}_{2},q_{3},....{\hat{q}}_{6}\right)+m_{33}^{-1}\left(\tau_{f3}-F_{r3}\right)+\rho_{6} \end{array}\} \end{array}$$ (44)

Where ${\hat{q}}_{1}$ is the estimated position and ${\hat{q}}_{2}$ is corresponding velocity of waist joint. Likewise, ${\hat{q}}_{3}$, ${\hat{q}}_{5}$ are estimated positions and ${\hat{q}}_{4}$, ${\hat{q}}_{6}$ are the estimated velocities for, shoulder and elbow joints respectively. The *ρ*_1_, *ρ*_2_, *ρ*_3_, *ρ*_4_, *ρ*_5_ and *ρ*_6_ shows the correction term for state vectors of robot manipulator joint. The Correction terms are defined as,

$$\begin{array}{c} \begin{array}{cc} \rho_{1} & =\alpha_{1}{\left|q_{1}-{\hat{q}}_{1}\right|}^{1/2}sign\left(q_{1}-{\hat{q}}_{1}\right)\\ \rho_{2} & =\beta_{1}\text{sign}\left(q_{1}-{\hat{q}}_{1}\right)\\ \rho_{3} & =\alpha_{2}{\left|q_{3}-{\hat{q}}_{3}\right|}^{1/2}sign\left(q_{3}-{\hat{q}}_{3}\right)\\ \rho_{4} & =\beta_{2}\text{sign}\left(q_{3}-{\hat{q}}_{3}\right)\\ \rho_{5} & =\alpha_{3}{\left|q_{5}-{\hat{q}}_{5}\right|}^{1/2}sign\left(q_{5}-{\hat{q}}_{5}\right)\\ \rho_{6} & =\beta_{3}\text{sign}\left(q_{5}-{\hat{q}}_{5}\right) \end{array}\} \end{array}$$ (45)

where *α*_1_ and *β*_1_ are constant design parameters. The torque input of robot manipulator is given to both models i.e. the estimated and actual models. The control law is applicable by applying the estimated velocities to the robot manipulator model with unobservable velocities. The control input for given system is torque, therefore the input torque for waist joint, shoulder and elbow joints are referred from above equations are given below

$$\begin{array}{c} \tau_{f1}=m_{11}\left(-c_{1}z_{1}-e_{1}-k_{1}{\hat{q}}_{2}+k_{1}{\dot{q}}_{d1}+{\ddot{q}}_{d1}+F_{r1}\right)-{\hat{f}}_{1} \end{array}$$ (46)

$$\begin{array}{c} \tau_{f2}=m_{22}\left(-c_{2}z_{2}-e_{2}-k_{2}{\hat{q}}_{4}+k_{2}{\dot{q}}_{d2}+{\ddot{q}}_{d2}+F_{r2}\right)-{\hat{f}}_{2} \end{array}$$ (47)

$$\begin{array}{c} \tau_{f3}=m_{22}\left(-c_{3}z_{3}-e_{3}-k_{3}{\hat{q}}_{6}+k_{3}{\dot{q}}_{d3}+{\ddot{q}}_{d3}+F_{r3}\right)-{\hat{f}}_{3} \end{array}$$ (48)

The second phase in implementing sensor FTC is the residuals evaluation and they are created by the actual and estimated positions difference. Therefore, these residuals are evaluated through decision making block which determines the existence of a fault.

$$\begin{array}{c} R_{i}=Residuals=q_{i}-{\hat{q}}_{i} \end{array}$$ (49)

where *i* = 1, 3, 5.

**Remark**: The foremost step in sensor FTC is regarding switching of sensor. Every time a residual suggests a fault in the sensor attached to the system, the backup sensor automatically turns on thus handling the fault while providing the feedback.

## 4 Results and discussion

**Fig 9:**
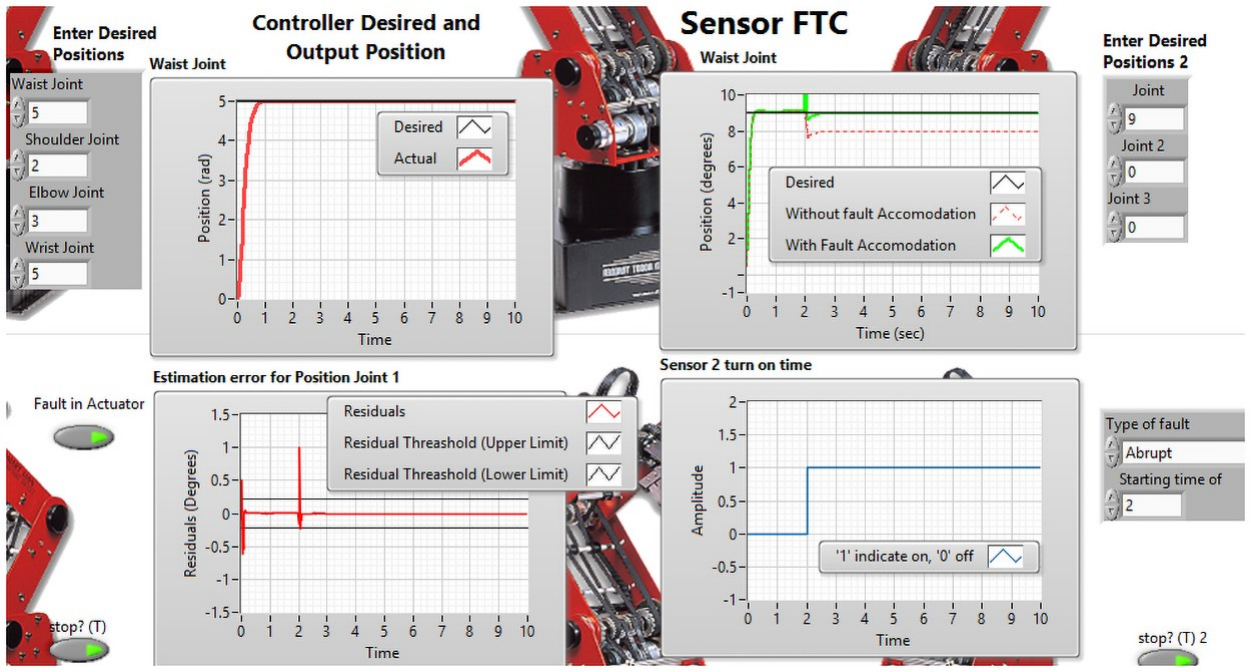
GUI of FTC.

In order to validate the effectiveness of the backstepping technique, sensor and actuator FTC algorithm, ED7220 robot model is used. The FTC algorithm has been simulated using LabView 2019/Matlab 2020. Fig 9 represents the graphical user interface(GUI) which is front panel of LabVIEW.

**Fig 10:**
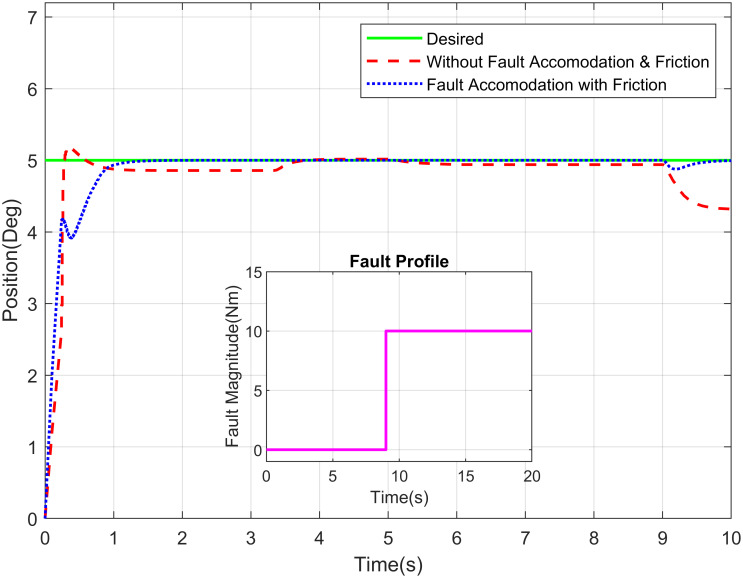
Position tracking of elbow joint of a robot manipulator with abrupt fault profile.

**Fig 11:**
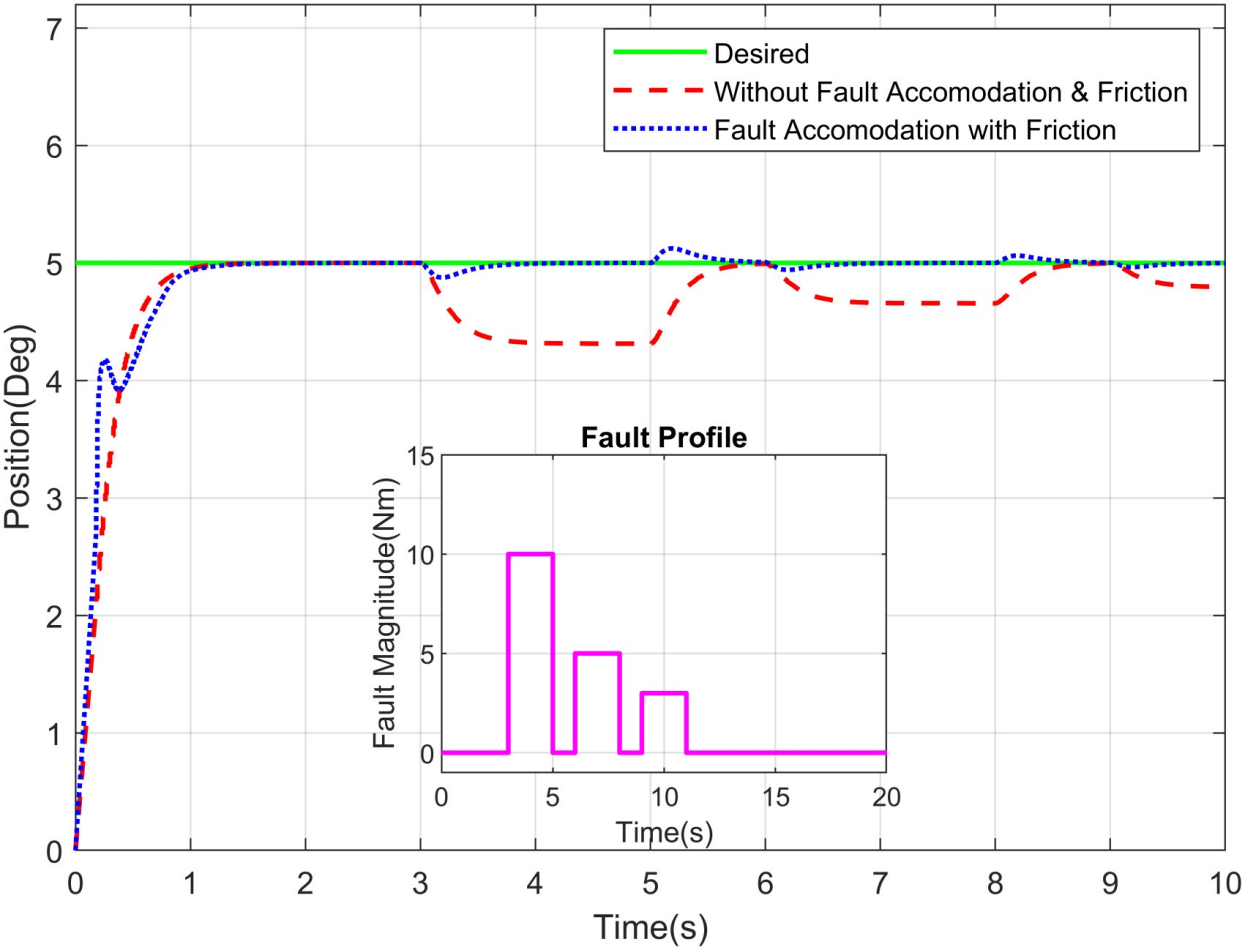
Position tracking of shoulder joint of a robot manipulator with intermittent fault profile.

The desired link position and type of fault are the inputs of this GUI, whereas actual link position is the output represented graphically in the GUI. The approaches developed in this research deal with sensor, actuator and/or component faults. The fault are events that can occur in various parts due to complex system dynamics and sophisticated hardware structures. For the estimation of actuator fault and tolerance, the abrupt type of fault is added on actuator of elbow joint at 8 seconds as shown in the Fig 10. It illustrates the proposed methodology for elbow joint of robot manipulator is sustaining the stability in existence of abrupt fault. Fig 11 describes the position tracking of robot manipulator shoulder joint with intermittent fault. The intermittent fault starting at 3 seconds in shoulder joint of robot manipulator which is effecting the system performance but FTC methodology is accommodating the intermittent fault with better stability.

**Fig 12:**
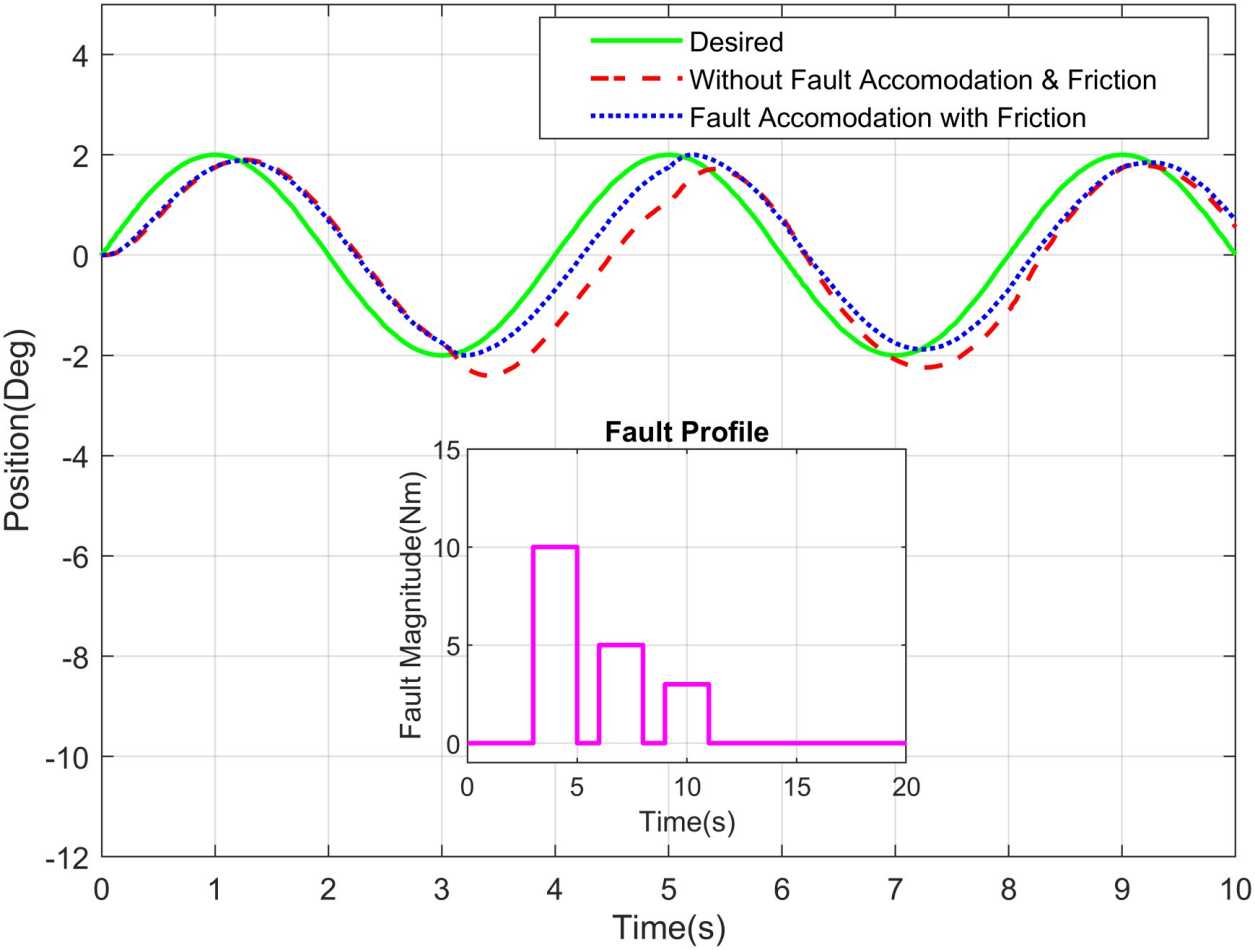
Sinusoidal response of elbow joint of a robot manipulator with intermittent fault profile.

**Fig 13:**
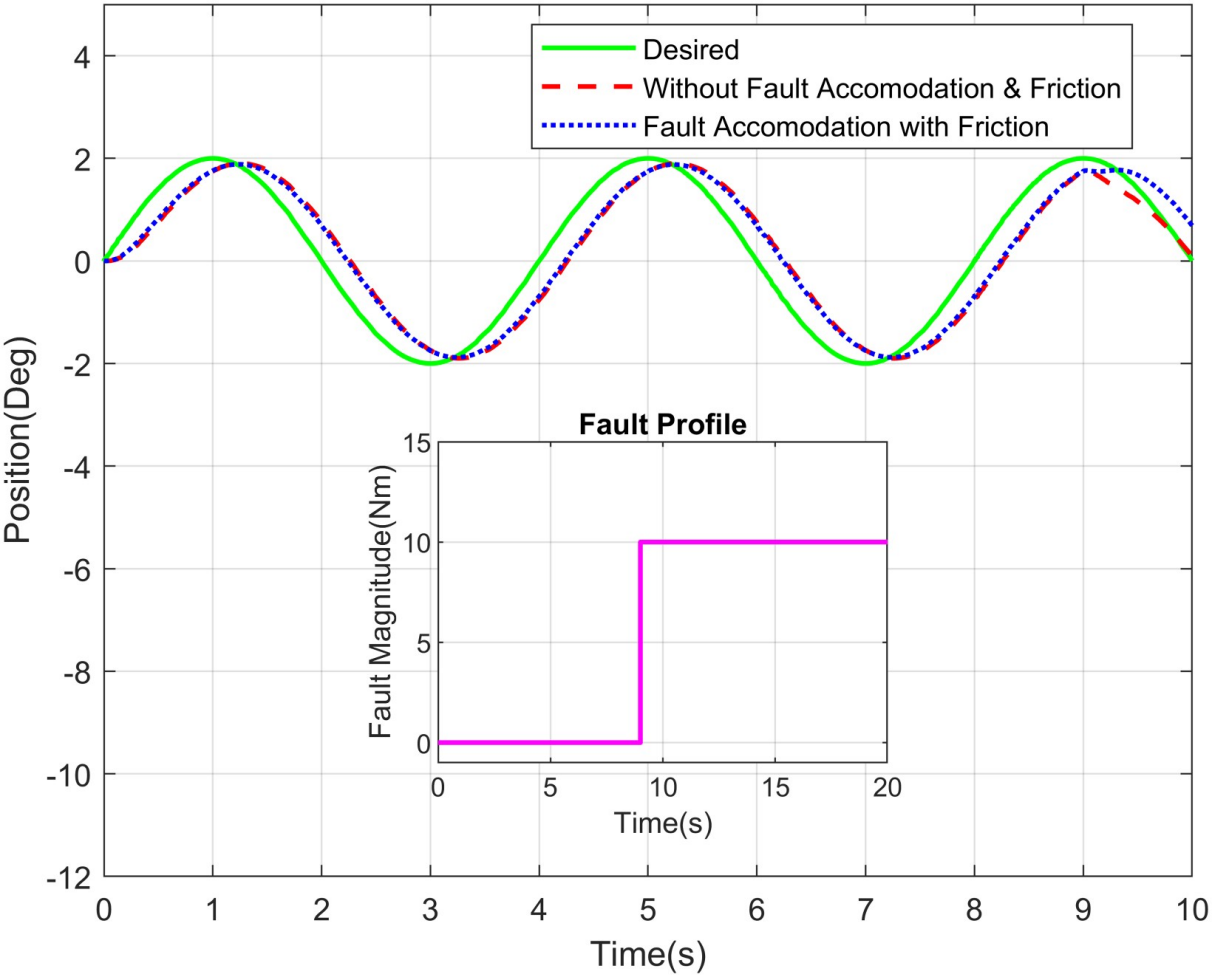
Sinusoidal response of shoulder joint of a robot manipulator with abrupt fault profile.

Similarly, the Figs 12 and 13 demonstrate the sinusoidal response of shoulder joint and elbow joint respectively with their fault profiles. The abrupt, intermittent, and incipient are the generally present kind of faults in the sensor therefore such faults are considered for simulation purpose. The intermittent fault appears in the waist joint of robot manipulator having optical sensor at two seconds. The incipient and abrupt fault occurs at five second for shoulder and waist joints, respectively.

**Fig 14:**
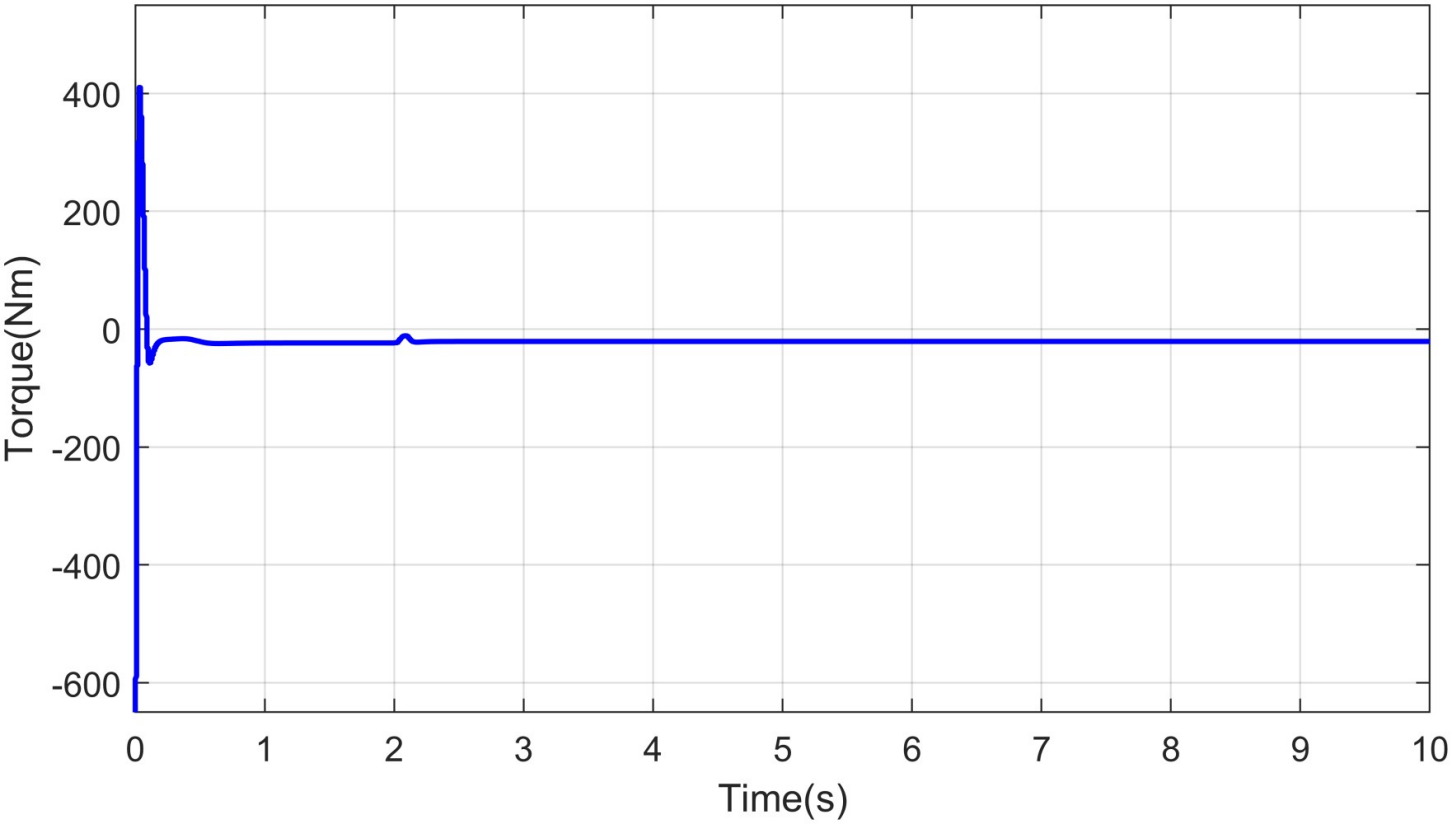
Control effort of waist joint of a robot manipulator.

The control effect is depicted in the form of applied torque to the waist joint in Fig 14 with abrupt fault at five seconds. Residuals profile can be helpful to detect the type of fault in the robot system, the residuals upper and lower limit is set to 0.22 for shoulder joint of robot manipulator. When there is no existence of fault then the residual signal is almost zero.

**Fig 15:**
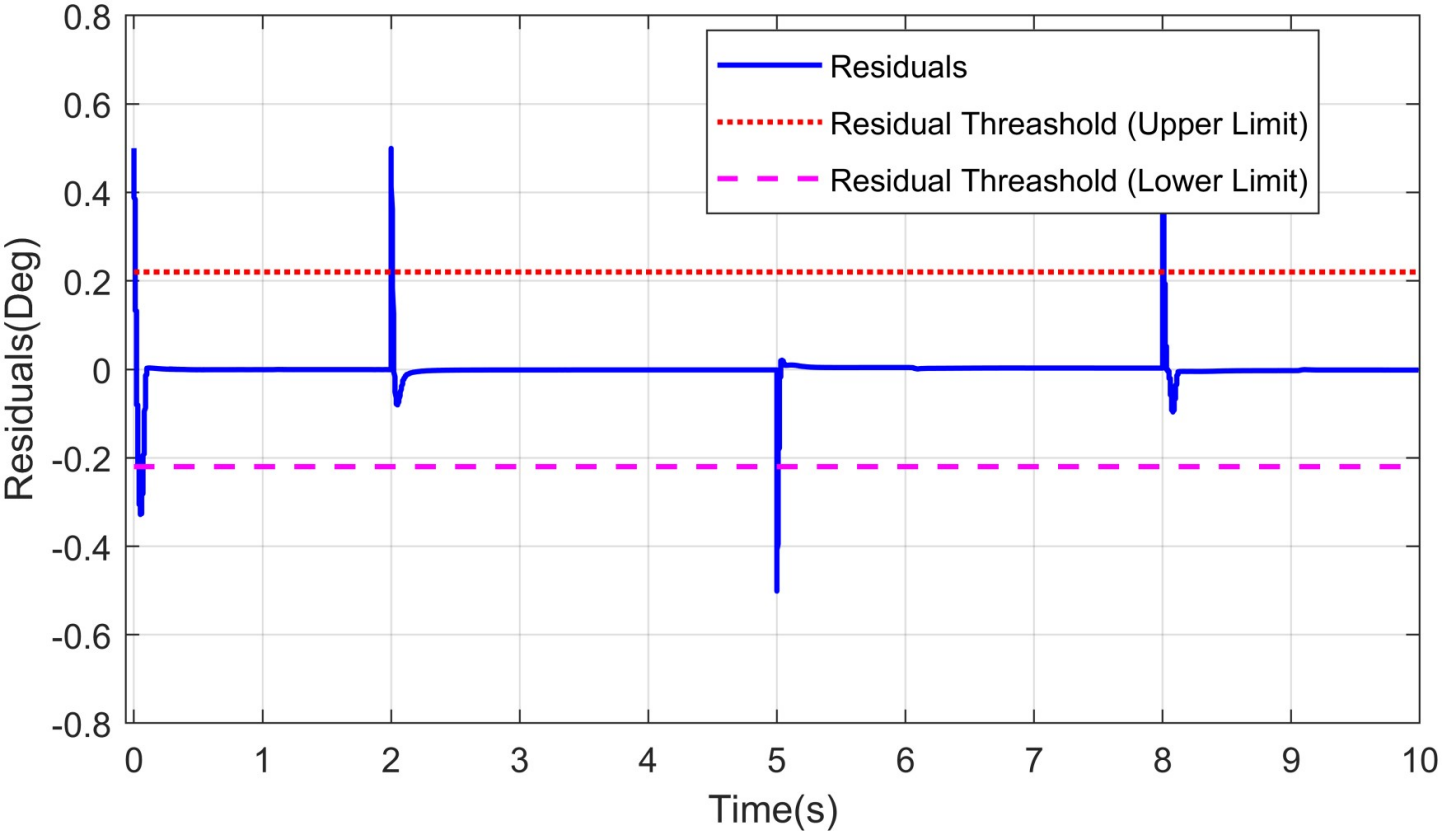
Residuals for fault profile in robot manipulator joint.

The indication of fault through residuals demonstrates only when the threshold limits are surpassing by residuals. For tolerating the fault, the feedback signal is provided by redundant sensor for waist joint which turns on instantly and same methodology can be employed to other robot manipulator joints with multi DoF movement. Residuals are determined by the comparison of actual positions with positions predicted by the observers. These residuals are then transferred to the decision-making block for assessment. The existence of a fault can be calculated by evaluation. As a result, the fault estimation block estimates the fault’s type and magnitude, and the nominal control rule is reconfigured to modify the response in the presence of the fault. Residuals with upper and lower threshold limits are also shown in Fig 15.

**Fig 16:**
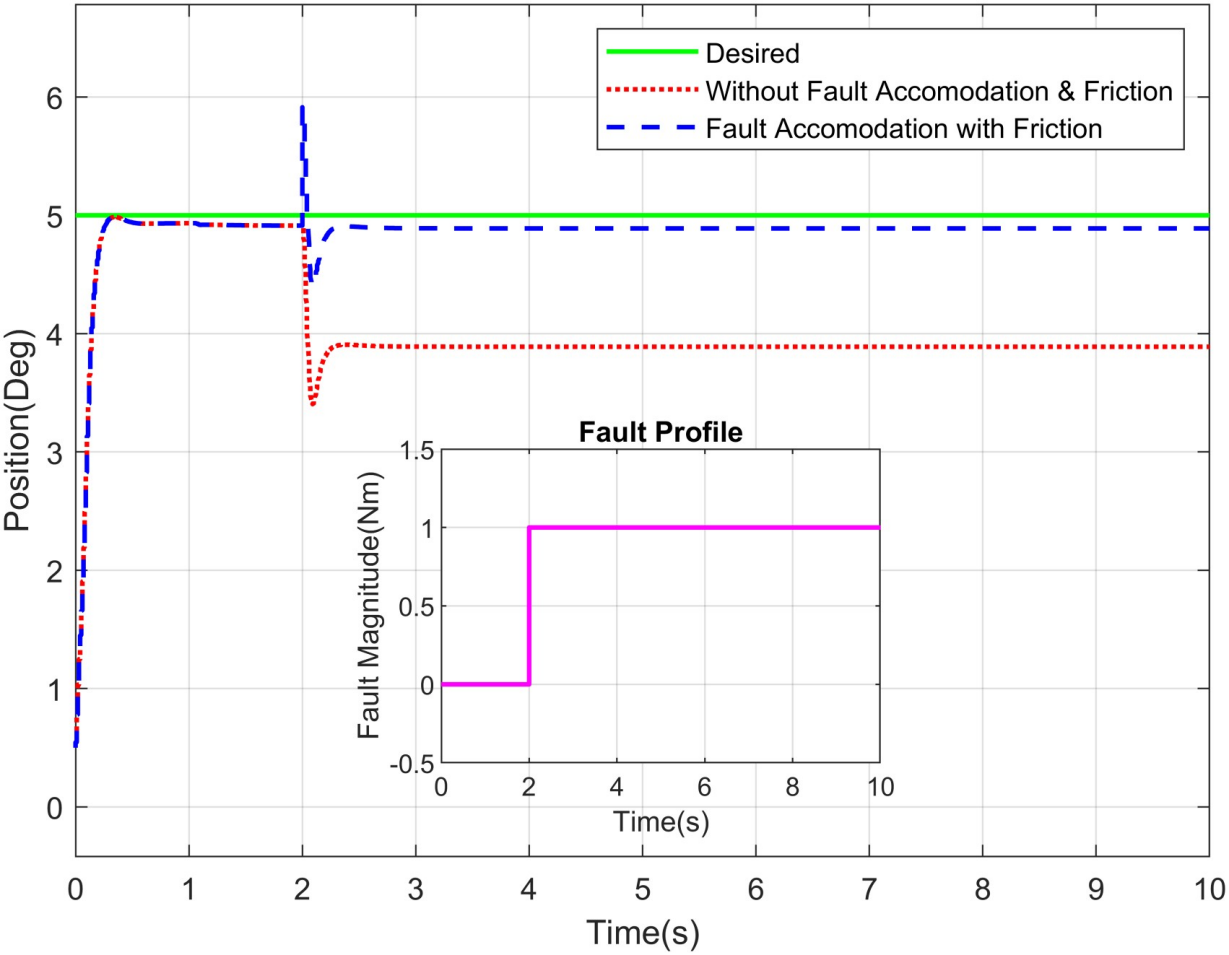
Position tracking of waist joint of a robot manipulator with abrupt fault profile.

**Fig 17:**
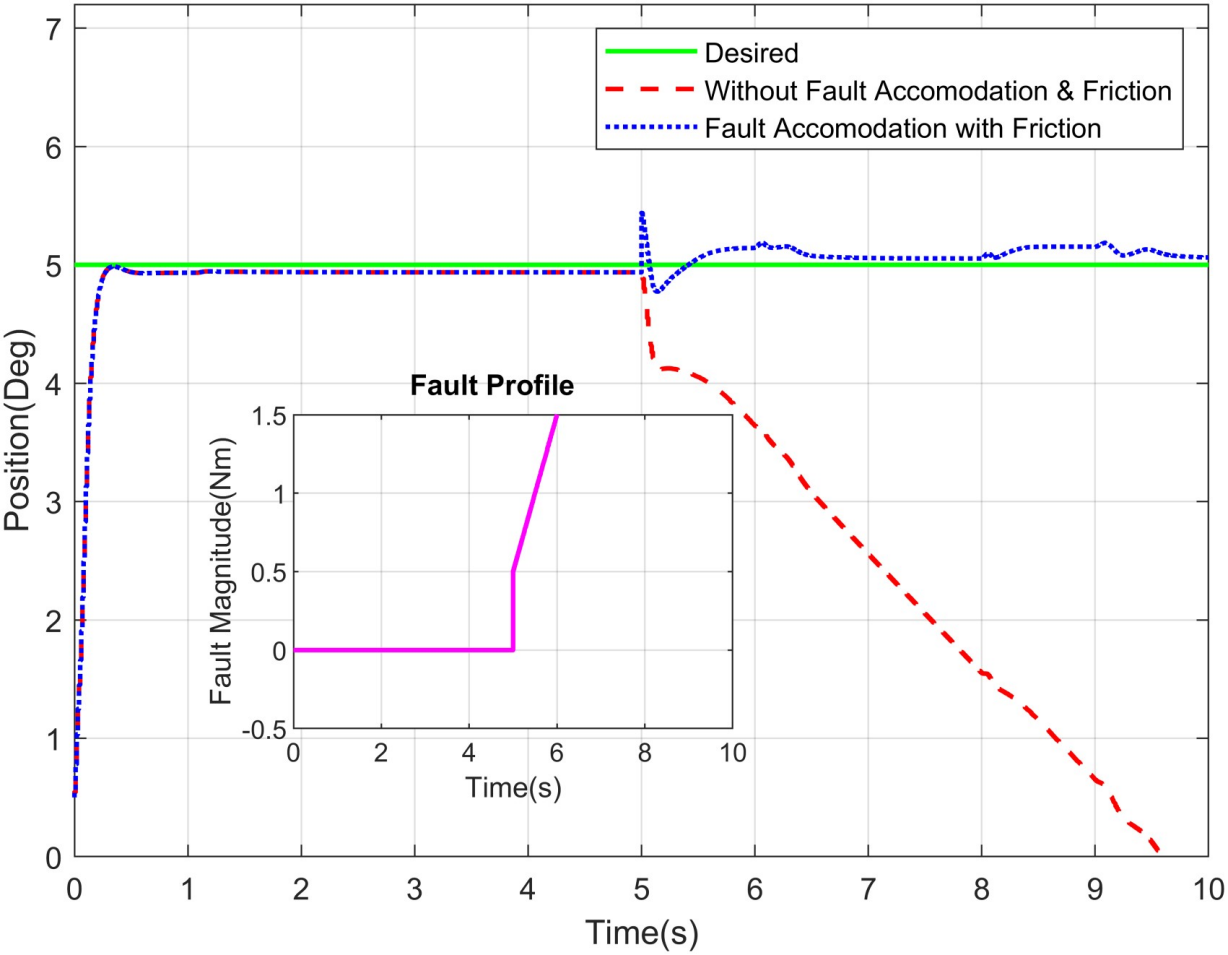
Position tracking of elbow joint of a robot manipulator with incipient fault profile.

**Fig 18:**
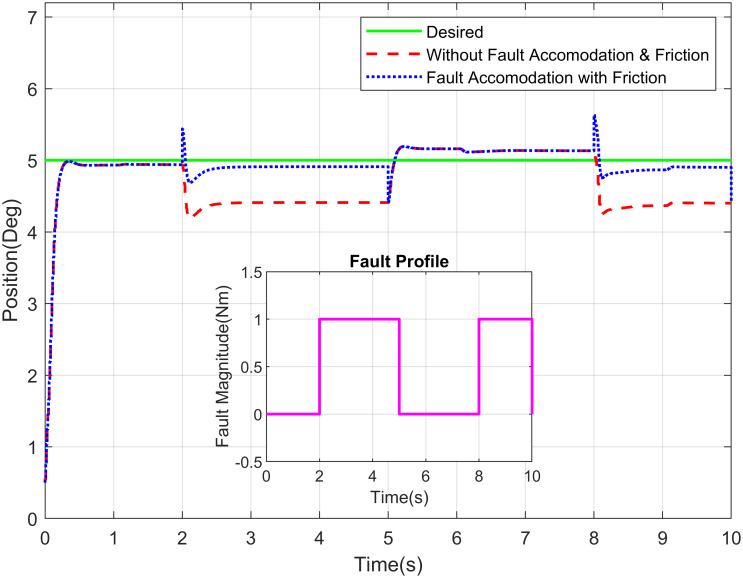
Position tracking of shoulder joint of a robot manipulator with intermittent fault profile.

The Figs 16–18 demonstrate the tracking performance with accommodation of fault when friction between the moving surfaces is considered and when it is ignored (for simplicity). These results are carried out on waist, shoulder and elbow joints, respectively. The assessment of required response with and without fault of sensor is shown in these figures. The non-existence of FTC of sensor suggests that the switching sensor is not available and the faulty sensor signal offers the feedback. Therefore, there is degradation in tracking performance in that scenario whereas in the incident with sensor switching, it gives better efficiency and performance nearly closes to the free scenario of fault free. The proposed system is very well coupled so, addition of fault to joint will affect the tracking performance of joints.

## 5 Conclusion

Industrial robots are employed to accomplish sensitive tasks. These robot manipulators are designed to tolerate faults up to some extent, in order to guarantee the dependability, safety and reliability. The faults and effects of friction are predominantly critical for robot manipulator. The actuator and sensor FTC are proposed in this article for ED-7220C robot manipulator considering with friction using dyanmic model. FTC gives some supplement control to compensate for faults and defects that may possibly take place in a system. Actuator FTC technique is established on adaptive back-stepping method to estimate the fault in the system. The methodology is made robust to actuator faults. Henceforth for the random actuator fault, the control law is reconfigured depending on estimated fault profile. FTC controller thus monitors and modifies itself and lessens the need of manual intervention. Similarly, the Sensor’s FTC design method is established. The fault in the system is found by considering the difference among the initial sensor signal value and estimated value. The observer-based design is used to provide the fault estimates through redundant sensor. Simulation results demonstrate the effectiveness of designed control algorithm that stabilizes the system in the existence of actuator and sensor faults for five DoF robot manipulator.
